# Topics of Public Interest

**Published:** 1910-09

**Authors:** 


### TOPICS OF PUBLIC INTEREST

### Women in Tuberculosis War

### Over a Million United to Fight Consumption in Every State

(Press Service of the National Association for the Study and Prevention of Tuberculosis)

What women have done in the last four years in the campaign against tuberculosis, is discussed in an interesting report issued August 18, 1910, by the National Association for the Study and Prevention of Tuberculosis.

Four years ago the only active women workers in the anti-tuberculosis movement were a little group of about thirty women's clubs. Today 800,000 women under the Health Department of the General Federation of Women's Clubs in every state and territory of the United States are banded together against this disease, and more than 2,000 clubs are taking a special interest in the crusade. Not less than $500,000 is raised annually by them for tuberculosis work, besides millions that are secured through their efforts in state and municipal appropriations. Mrs. Rufus P. Williams is the chairman of the department that directs this work.

In addition to the work of the General Federation of Women's Clubs, The Public Health Education Committee of the American Medical Association, composed largely of women physicians has carried on an educational campaign of lectures during the past year in which thousands have been reached. The Mothers' Congress, the Young Women's Christian Association, and many unattached clubs bring the number of women united in the tuberculosis war to well over a million. There is not a state in the union where some work has not been done.

Through the activity of women, sanatoria and hospitals for the treatment of tuberculosis have been erected; traveling libraries have been circulated, posters, circulars and other kinds of literature have been distributed to the number of millions of pieces; thousands of lectures have been given, large sums of money have been secured, hundreds of needy cases have been helped; tuberculosis work has been started in many communities where no movement had existed; and millions of women have learned the dangers and methods of prevention of tuberculosis.

The work of the women extends from the drawing room of the rich to the homes of the poor, and embraces all classes, including the factory girl and millionaire. During the coming year a special campaign of lectures to women will be carried on in all parts of the United States.

